# Author Correction: Enhancing catalytic performance of dilute metal alloy nanomaterials

**DOI:** 10.1038/s42004-020-0310-5

**Published:** 2020-05-11

**Authors:** Mathilde Luneau, Erjia Guan, Wei Chen, Alexandre C. Foucher, Nicholas Marcella, Tanya Shirman, David M. A. Verbart, Joanna Aizenberg, Michael Aizenberg, Eric A. Stach, Robert J. Madix, Anatoly I. Frenkel, Cynthia M. Friend

**Affiliations:** 1 Department of Chemistry and Chemical Biology, Harvard University, Cambridge, MA 02138 USA; 2 Department of Materials Science and Chemical Engineering, Stony Brook University, Stony Brook, New York, 11794 USA; 3 Department of Physics and School of Engineering and Applied Sciences, Harvard University, Cambridge, MA 02138 USA; 4 Department of Materials Science and Engineering, University of Pennsylvania, Philadelphia, PA 19104 USA; 5 John A. Paulson School of Engineering and Applied Sciences, Harvard University, Cambridge, MA 02138 USA; 6 Wyss Institute for Biologically Inspired Engineering, Harvard University, Cambridge, MA 02138 USA; 7 Division of Chemistry, Brookhaven National Laboratory, Upton, New York, 11973 USA

**Keywords:** Porous materials, Nanoparticles, Heterogeneous catalysis

Correction to: *Communications Chemistry* 10.1038/s42004-020-0293-2, published online 9 April 2020.

The original version of this Article omitted the following from the Acknowledgements:

This research used 8-ID (ISS) beamline of the National Synchrotron Light Source, a U.S. DOE Office of Science User Facility operated for the DOE Office of Science by Brookhaven National Laboratory (BNL) under Contract No. DE-SC0012704. Work was also carried out in part at the Singh Center for Nanotechnology at the University of Pennsylvania which is supported by the National Science Foundation (NSF) National Nanotechnology Coordinated Infrastructure Program grant NNCI-1542153. Additional support to the Nanoscale Characterization Facility at the Singh Center has been provided by the Laboratory for Research on the Structure of Matter (MRSEC) supported by the National Science Foundation (DMR-1720530).

This has now been corrected in both the PDF and HTML versions of the Article.

