# Supplementary material for: Enhancing catalytic performance of dilute metal alloy nanomaterials
Source: Commun Chem. 2020 Apr 9;3:46. doi: 10.1038/s42004-020-0293-2 (PMC9814734; doi:10.1038/s42004-020-0293-2)
Supplement: Supplementary file 1 — Supporting Information [file 42004_2020_293_MOESM1_ESM.pdf]

Supporting Information

Enhancing catalytic performance of dilute metal alloy nanomaterials

Mathilde Luneau[1], Erjia Guan[2], Wei Chen[3], Alexandre C. Foucher[4], Nicholas Marcella[2], Tanya Shirman[5,6], David M.A. Verbart[1], Joanna Aizenberg[1,5,6], Michael Aizenberg[6], Eric A. Stach[4], Robert J. Madix[5], Anatoly I. Frenkel[2,7], Cynthia M. Friend[1,5]

[1]Department of Chemistry and Chemical Biology, Harvard University, Cambridge, MA 02138, USA
[2]Department of Materials Science and Chemical Engineering, Stony Brook University, Stony Brook, New York 11794, USA
[3]Department of Physics and School of Engineering and Applied Sciences, Harvard University, Cambridge, MA 02138, USA
[4]Department of Materials Science and Engineering, University of Pennsylvania, Philadelphia, Pennsylvania 19104, USA
[5]John A. Paulson School of Engineering and Applied Sciences, Harvard University, Cambridge, MA 02138, USA
[6]Wyss Institute for Biologically Inspired Engineering, Harvard University, Cambridge, MA 02138, USA
[7]Division of Chemistry, Brookhaven National Laboratory, Upton, New York 11973, USA

Supplementary Table 1: Size of nanoparticles embedded in RCT-SiO_2 and atomic Pd concentration of $Pd_{0.04}Au_{0.96}$ RCT-SiO_2

	Nanoparticle average diameter (nm)[a]	atomic % Pd[b]	Total metal loading wt.%[b]
$Pd_{0.04}Au_{0.96}$	6.0 ± 1.6	4.3 ± 0.2	2.5

a. Average particle size as determined with TEM ; b. measured by ICP-MS

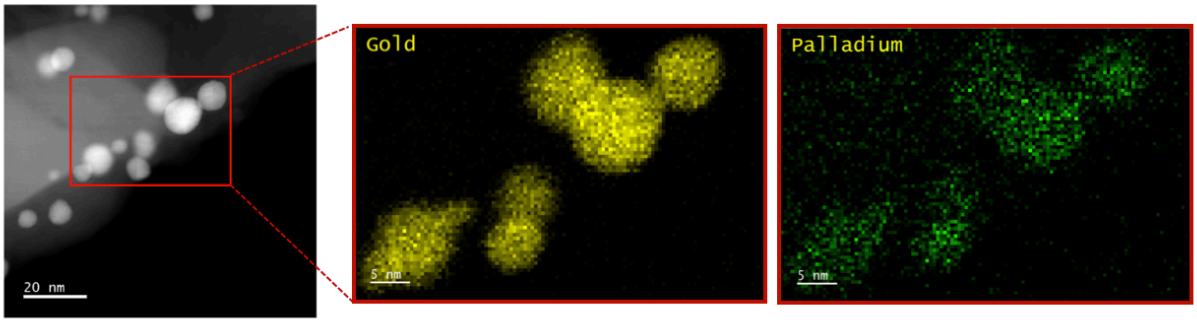

Supplementary Figure 1: TEM image and EDS maps showing the distribution of Pd and Au in $Pd_{0.04}Au_{0.96}$ RCT-SiO_2 after calcination in air for two hours at 773K

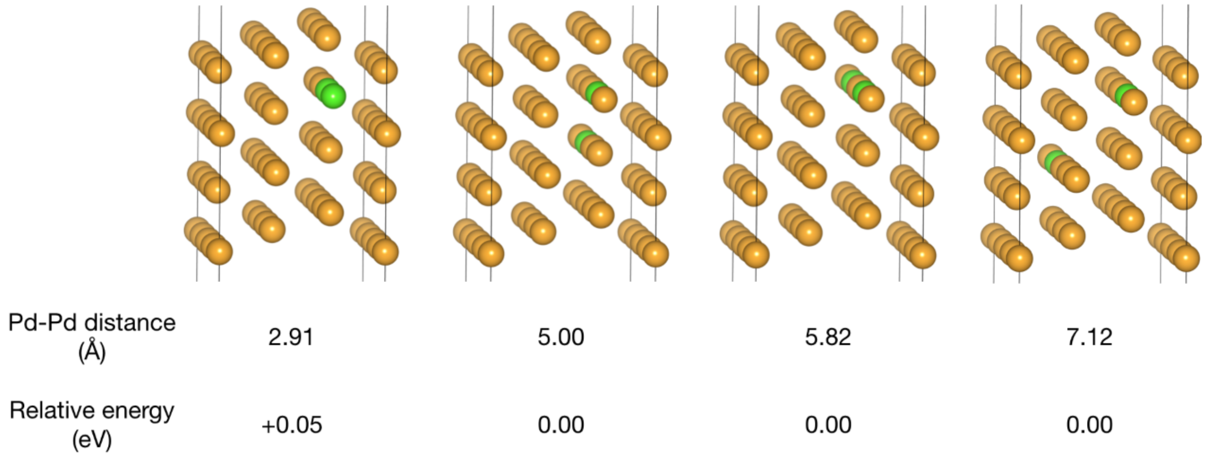

Supplementary Figure 2: DFT-calculated relative energies and Pd-Pd distances for a Au(211) system with 2 Pd atoms. The three lowest energy configurations have Pd atoms isolated, and are lower in energy than the configuration with two Pd next to each other in the lattice by 0.05 eV.

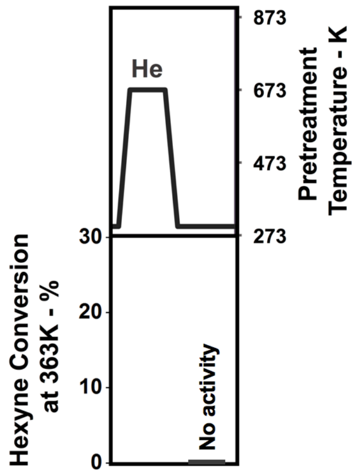

Supplementary Figure 3: $Pd_{0.04}Au_{0.96}$ RCT-SiO_2 is not active after treatment in He at 673K as shown by 1-hexyne conversion at 363K. He treatment : Patm ; 673K for 30 min ; ramp 10K min^{-1}; Total Flow rate = 40 mL min^{-1} ; Reaction: Patm ; 1% 1-hexyne ; 20% H_2 in He balance ; T = 363K ; mcat = 20 mg ; Total Flow rate = 50 mL min^{-1} ; $m_{catalyst}$ = 20 mg ; GHSV = 3,800 h^{-1}

Supplementary Table 2: Conversion of 1-hexyne and selectivity towards 1-hexene after treatments in O_2, CO and H_2 at different temperatures

**Treatment**	**Temperature K**	**1-Hexyne Conversion %**	**1-Hexene Selectivity %**
O_2	323	19 (0.3)	96 (1)
	523	23 (1)	95 (1)
	673	30 (0.3)	96 (1)
	773*	27 (1)	96 (0.2)
H_2	523	21 (1)	96 (1)
	673	6 (1)	100 (1)
CO	303	10 (0.3)	98 (1)
	473	11 (0.2)	95 (0.2)
	673	0	/

**not performed sequentially after the treatment at 673K*

Supplementary Table 3: Complete EXAFS analysis[a] of RCT silica supported $Pd_{0.04}Au_{0.96}$ nanoparticles after pretreatments of O_2, H_2 and CO.

Pre-treatment	**Pd–O**			**Pd–Au**			**Pd–Pd**			ΔE_0 (eV)
	CN	*R* (Å)	σ^2 (Å^2)	*CN*	*R* (Å)	σ^2 (Å^2)	*CN*	*R* (Å)	σ^2 (Å^2)	
O_2 (673 K)	0.2 (1)	2.20 (2)	0.005 (2)	11.5 (4)	2.825 (2)	0.0073 (3)	0.2 (1)	2.80 (2)	0.00 (2)	2.7 (1)
H_2 (673 K)	-	-	-	11.6 (5)	2.825 (2)		0.2 (1)	2.82 (2)		
CO (298 K)	-	-	-	11.3 (5)	2.825 (2)		0.2(1)	2.78 (2)		
O_2 (673 K, recover)	0.2 (1)	2.20 (2)	0.005 (2)	11.5 (5)	2.825 (2)		0.2 (1)	2.80 (2)		

[a]*CN*, coordination number; *R*, distance between absorber and backscatterer atoms; σ^2, disorder term (EXAFS Debye-Waller factor); ΔE_0, inner potential correction.

Note: All data presented in the table were collected at 298 K after each pretreatment. In the fitting of FEFF6 theory to the data, the Pd–O, Pd–Au and Pd–Pd coordination numbers, corrections to the model bond distances, corrections to the photoelectron energy origin were varied. Amplitude reduction factors S_0^2 (0.87 ± 0.02) were obtained from the EXAFS data fits for Pd foil spectrum and were fixed to those values for the determination of coordination numbers in the $Pd_{0.04}Au_{0.96}$ RCT-SiO_2. To minimize the uncertainties, the data for several regimes were analyzed concurrently by applying several constraints between fitting parameters: the inner potential correction value was fixed to all models; the Debye-Waller factors were fixed for the shells with the same scatterer after each pretreatment; the bond distances were fixed for the Pd-Au shell after each pretreatment.

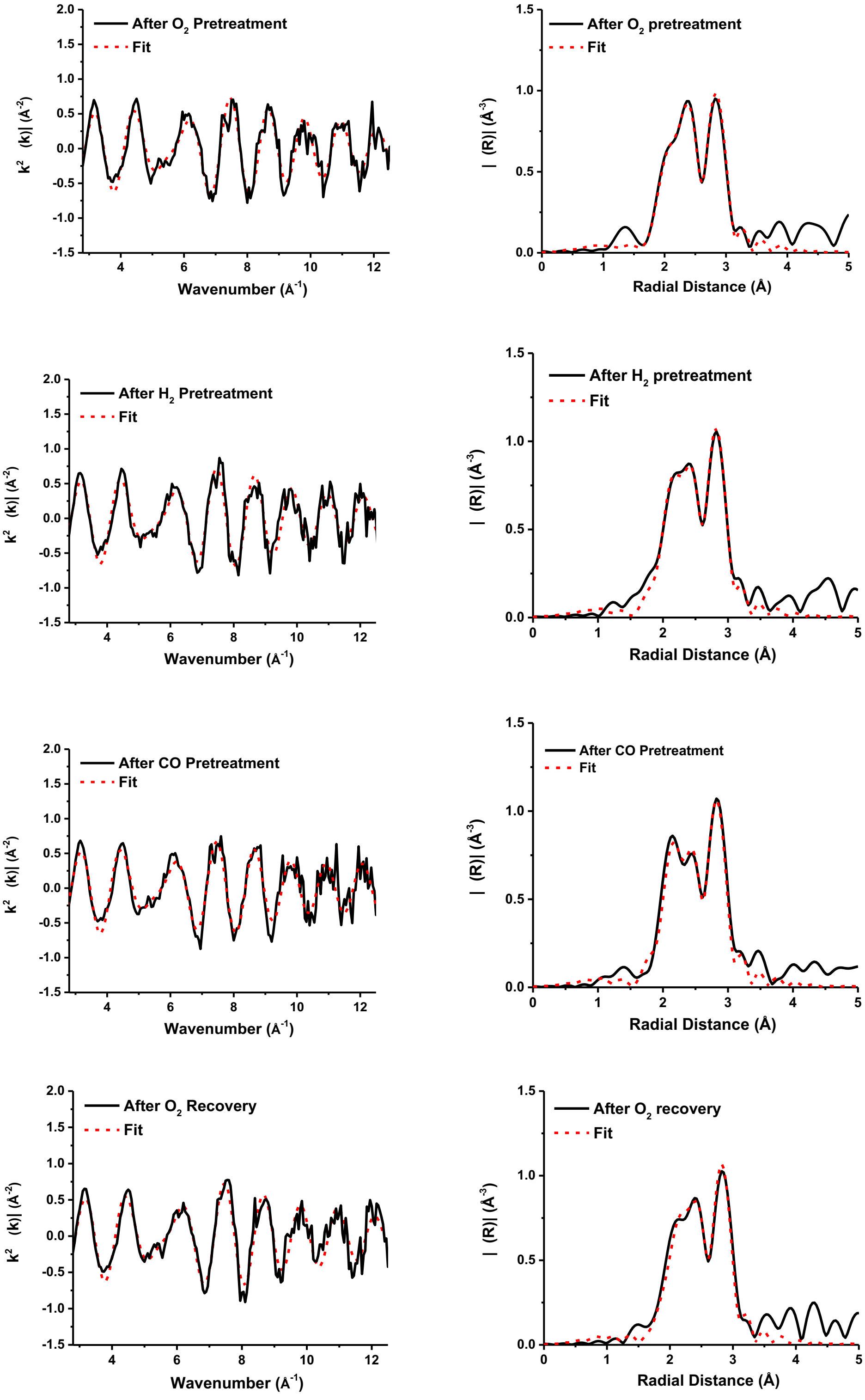

Supplementary Figure 4 : EXAFS data and theoretical fits for $Pd_{0.04}Au_{0.96}$ RCT-SiO_2 after O_2, H_2, CO pretreatments and O_2 recovery: k^2-weighted EXAFS function, $k^2(\chi)$ (solid line) and theoretical fit (dashed line); magnitude of the Fourier transform of the k^2-weighted data (solid line) and theoretical fit (dashed line) of the samples.

Estimation of Pd atoms at the surface from EXAFS quantitative fitting
Supplementary Equation 1

$$\%\ Pd\ atoms\ at\ the\ surface = \frac{CN\ (Pd-O)Pd_{0.04}Au_{0.96}}{CN\ (Pd-O)Surface\ PdO\ (1\ layer)} = \frac{0.2}{1} = 20\ \%$$

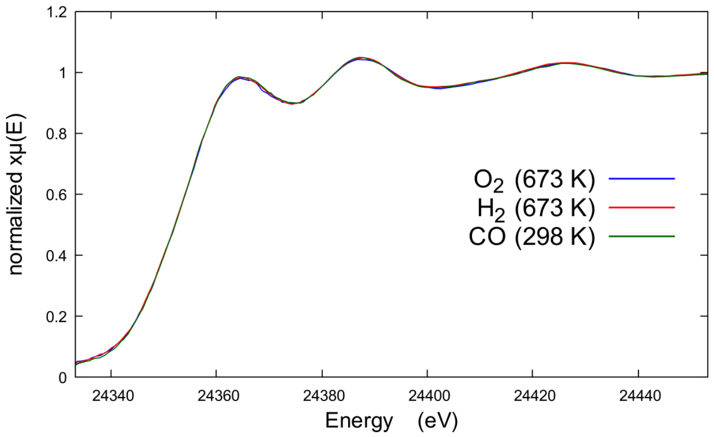

Supplementary Figure 5: Experimental XANES at the Pd K-edge of $Pd_{0.04}Au_{0.96}$ RCT SiO_2 after treatment in O_2 at 673 K (blue), H_2 at 673K (red) and CO at 298 K (green)

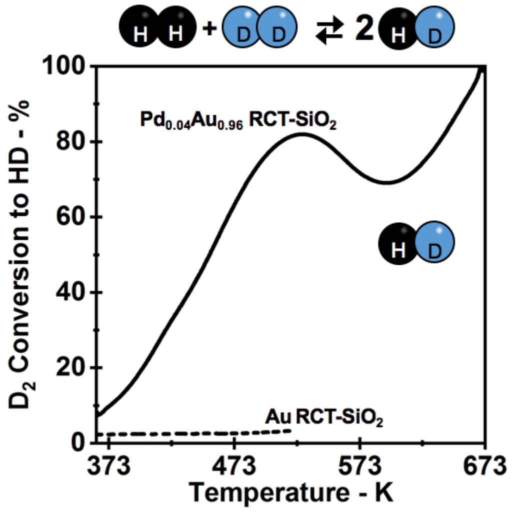

Supplementary Figure 6: HD formation occurs when $Pd_{0.04}Au_{0.96}$ RCT-SiO_2 is exposed to a mixture of D_2 and H_2 after pretreatment in oxygen at high temperature. Control experiment on pure Au RCT-SiO_2 shows no formation of HD. HD exchange reaction: 20% H_2; 1% D_2 in He balance over $Pd_{0.04}Au_{0.96}$ RCT-SiO_2; 10% H_2; 10% D_2 over pure Au RCT-SiO_2; 10K min^{-1} ramp ; m_{cat} = 20 mg ; Total Flow rate = 50 mL min^{-1} ; GHSV = 3,800 h^{-1}

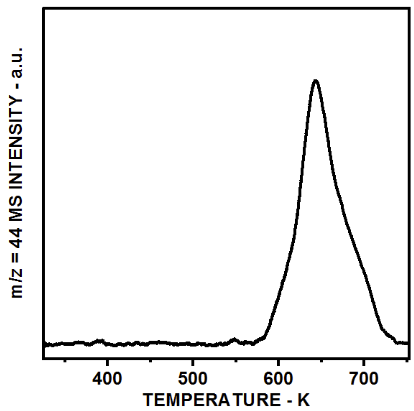

Supplementary Figure 7: Carbon deposition occurs after pretreatment in CO at 673K over $Pd_{0.04}Au_{0.96}$ RCT-SiO_2 as demonstrated by the formation of CO_2 starting at ~600K monitored with m/z = 44 by online MS during temperature programmed oxidation (TPO) performed after oxygen treatment at 773K directly followed by CO treatment at 673K without exposure to reaction conditions. Oxygen pretreatment: Patm ; 20% O_2/He at 773K for 30 min ; ramp 10K min^{-1}; Total Flow rate = 50 mL min^{-1} ; CO pretreatment: Patm ; 10% CO/He at 673K for 30 min ; ramp 10K min^{-1} Total Flow rate = 50 mL min^{-1} ; TPO : Patm ; 20% O_2/He at 773K for 30 min ; ramp 10K min^{-1}; Total Flow rate = 20 mL min^{-1}

Supplementary Table 4: Results of EXAFS analysis[a] of RCT silica - supported $Pd_{0.04}Au_{0.96}$ nanoparticles under H_2 and 1-Hexyne at 363 K after pretreatments of O_2 and H_2

Pre-Treatment	**Pd–O**			**Pd–Au**			**Pd–Pd**			ΔE_0 (eV)
	CN	*R* (Å)	σ^2 (Å^2)	*CN*	*R* (Å)	σ^2 (Å^2)	*CN*	*R* (Å)	σ^2 (Å^2)	
O_2 (673 K)	-	-	-	10.7 (7)	2.813 (5)	0.0087 (8)	0.2 (2)	2.70 (3)	0.000 (4)	- 0.5 (3)
H_2 (673 K)	-	-	-	10.4 (7)	2.813 (5)		0.4 (3)	2.77 (2)		

[a]*CN*, coordination number; *R*, distance between absorber and backscatterer atoms; σ^2, disorder term (EXAFS Debye-Waller factor); ΔE_0, inner potential correction.

Note: All data presented in the table were collected at 363 K after each pretreatment. In the fitting of FEFF6 theory to the data, the Pd–O, Pd–Au and Pd–Pd coordination numbers, corrections to the model bond distances, corrections to the photoelectron energy origin were varied. Amplitude reduction factors S_0^2 (0.78 ± 0.02) were obtained from the EXAFS data fits for Pd foil spectrum and were fixed to those values for the determination of coordination numbers in the $Pd_{0.04}Au_{0.96}$ RCT-SiO_2. The fitting ranges in k-space and r-space were between 2 to 11.4 Å^{-1} and 1.5 to 3.3 Å, respectively. The fits were done by using k-weights of 1 and 2 concurrently. To minimize the uncertainties, both data were analyzed concurrently by applying several constraints between fitting parameters: the inner potential correction value was fixed to all models; the Debye-Waller factors were fixed for the shells with the same scatterer after each pretreatment; the bond distances were fixed for the majority bond (Pd-Au) shell after each pretreatment.

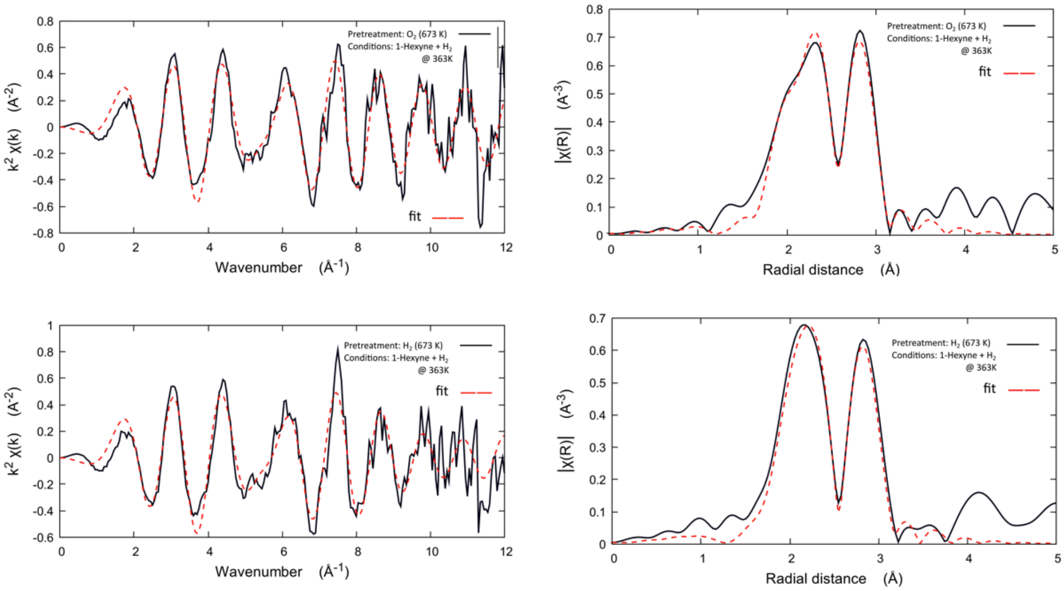

Supplementary Figure 8: Pd K-edge EXAFS data and theoretical fits for $Pd_{0.04}Au_{0.96}$ RCT-SiO_2 in reaction conditions (H_2 and 1-Hexyne) after O_2 (top) and H_2 (bottom) pretreatments: k^2-weighted EXAFS function, k^2 χ (k) (solid line) and theoretical fit (dashed line), left panels ; and magnitude of the Fourier transform of the k^2-weighted data (solid line) and theoretical fit (dashed line) of the samples right panels.
